# Plastic ingestion by freshwater turtles: a review and call to action

**DOI:** 10.1038/s41598-021-84846-x

**Published:** 2021-03-23

**Authors:** Adam G. Clause, Aaron J. Celestian, Gregory B. Pauly

**Affiliations:** 1 Urban Nature Research Center and Department of Herpetology, Natural History Museum of Los Angeles County, Los Angeles, CA USA; 2 Department of Mineral Sciences, Natural History Museum of Los Angeles County, Los Angeles, CA USA

**Keywords:** Herpetology, Conservation biology

## Abstract

Plastic pollution, and especially plastic ingestion by animals, is a serious global issue. This problem is well documented in marine systems, but it is relatively understudied in freshwater systems. For turtles, it is unknown how plastic ingestion compares between marine and non-marine species. We review the relevant turtle dietary literature, and find that plastic ingestion is reported for all 7 marine turtle species, but only 5 of 352 non-marine turtle species. In the last 10 years, despite marine turtles representing just 2% of all turtle species, almost 50% of relevant turtle dietary studies involved only marine turtles. These results suggest that the potential threat of plastic ingestion is poorly studied in non-marine turtles. We also examine plastic ingestion frequency in a freshwater turtle population, finding that 7.7% of 65 turtles had ingested plastic. However, plastic-resembling organic material would have inflated our frequency results up to 40% higher were it not for verification using Raman spectroscopy. Additionally, we showcase how non-native turtles can be used as a proxy for understanding the potential for plastic ingestion by co-occurring native turtles of conservation concern. We conclude with recommendations for how scientists studying non-marine turtles can improve the implementation, quality, and discoverability of plastic ingestion research.

## Introduction

Plastic pollution is a major global problem^1,2^. This pollution can cause environmental harm in several ways, but plastic ingestion by animals is especially common^2^. Almost 600 marine vertebrate species have been documented ingesting plastic^3^. This dietary plastic is often reported or suspected to cause animal mortality through obstruction, laceration, or perforation of the gastrointestinal tract^2^. Although more difficult to study, potential sublethal effects of plastic ingestion include malnutrition, immune impairment, and chemical contamination^4,5^. While many studies explore plastic ingestion impacts on individual animals, population-level effects on marine vertebrates remain largely unassessed^2^.

Multiple pathways introduce plastics into marine systems, but inputs from land-based activities are generally considered leading sources. These sources also deposit plastics into freshwater systems^6^. Given the well-documented negative individual-level impacts of plastic pollution on marine organisms, freshwater organisms are likely to be similarly affected—assuming that similar amounts of plastic exist in marine and freshwater habitats. However, both plastic volume and impacts to wildlife in freshwater systems remain comparatively understudied^6–9^.

The potential for plastic to negatively affect freshwater animals has serious conservation implications. Freshwater ecosystems are biologically diverse and already face multiple, interacting threats^10,11^. Leading stressors include flow modification, invasive species, climate change, degraded water quality, and habitat degradation in adjacent uplands^11,12^. This suite of threats has caused major worldwide declines in many freshwater taxa including fish, mammals, reptiles, amphibians, molluscs, and crayfish^13–15^. Nonetheless, better understanding of how plastic pollution affects freshwater systems is essential for accurately evaluating the imperilment of animals that live in these habitats.

One particularly at-risk group of freshwater animals are turtles. Over 50% of all recognized turtle species are considered threatened on the International Union for Conservation of Nature’s Red List of Threatened Species^16^, making them among the most imperiled animal groups on Earth. Habitat degradation and overexploitation drive most of this imperilment^17^, but many threats endanger turtles. The threat of plastic ingestion is well documented in all seven marine turtle species, with both lethal and sublethal effects reported. Marine turtles worldwide regularly ingest both macroplastics (> 5 mm) and microplastics (< 5 mm), spanning a wide range of plastic types. For additional details, see recent comprehensive coverage of marine turtles and plastic ingestion^2,5,18–20^.

Critically, for the 352 non-marine turtle species, some 75% of which inhabit freshwater^21^, plastic or human litter ingestion is rarely reported and has never been comprehensively reviewed. Given that many freshwater turtle species encounter plastic pollution, especially in or near urbanized habitats^22,23^, plastic ingestion could be a relevant yet underestimated conservation concern for these animals.

Plastic ingestion frequency in wild turtles is also understudied. Publications on turtle plastic ingestion are usually either anecdotal one-off observations, or involve mostly dying or dead turtles due to justifiable concerns about lethal sampling of these globally endangered taxa. As noted in a recent review^24^, this research limitation has led to a scarcity of comparatively unbiased data on plastic ingestion frequency in wild turtles.

Here, we offer the first comprehensive review of plastic and human litter ingestion by non-marine turtles, and compare the number of relevant diet studies (i.e., studies that could potentially detect ingested plastics) on non-marine turtles to those on marine turtles. Additionally, we present one of the few existing datasets on plastic ingestion frequency in wild turtles. This dataset involves a non-native population of the Red-eared Slider, *Trachemys scripta elegans*, in California, USA, which serves as a proxy for the potential threat of plastic ingestion faced by the sympatric Northwestern Pond Turtle, *Emys marmorata* (= *Actinemys marmorata* of some authors), which is declining range-wide^25^. We conclude with a call to action for dedicated study of plastic ingestion in non-marine turtles, and offer recommendations for best-practice standards for documenting and reporting plastic ingestion.

## Methods

### Literature review

We used ISI Web of Science to identify diet studies that did, or had the potential to, document plastic ingestion by free-living turtles. We reviewed two bodies of literature. First, we compiled all journal articles examining the diet of non-marine turtles published prior to January 2021, to review existing data on plastic ingestion in these animals. Second, we compiled all journal articles published in the past 10 years (from January 2010–December 2020) on the diet of both marine and non-marine turtles, to compare the relative research attention for these two animal groups. Throughout, we restricted our review to articles that involved turtle gastrointestinal tract dissection, stomach flushing, or fecal analysis, such that authors either identified ingested plastics or had the potential to identify such items. We excluded articles that only involved observational diet behavior by a turtle (e.g., notes documenting turtle[s] eating previously unreported prey species), because we deemed these to lack potential for documenting plastic ingestion. Similarly, we excluded studies that lacked direct behavioral observation or sampling of turtles, studies of captive turtles with controlled diets, and studies involving only stable-isotope, microbiota, or eDNA analyses. We also excluded reports of human food items such as discarded meat or fruit consumed by turtles. Because turtle dietary studies routinely report inorganic materials if present (typically sediment, sand, or pebbles), we assumed that such studies with the “potential” to identify ingested plastic would have done so if plastic was observed.

In our search, we used “turtle*” and “diet*” as topic terms, with the asterisks directing ISI Web of Science to return papers with the words “turtle,” “turtles,” “diet,” “diets,” or “dietary.” We then filtered the returned results for relevance by examining paper titles and abstracts. For each identified non-marine turtle diet study, we queried for the terms “plastic,” “litter,” “debris,” and “trash” using PDF search functions, and read the relevant sections of the Results and Discussion. We cross-referenced our ISI Web of Science sources by examining relevant natural history notes published in the journal *Herpetological Review*, and finally combined the ISI Web of Science and *Herpetological Review* results into two master lists. One additional relevant article on plastic ingestion by a freshwater turtle was brought to our attention by colleagues. We recognize 359 extant turtle species globally of which seven are sea turtles^21,26–28^, while acknowledging that taxonomy in this group remains fluid.

### Field survey

Our study site was the University of California, Davis Arboretum waterway (hereafter, UCD Arboretum) in Yolo County, California, USA (38.53°N, 121.76°W). This semi-urbanized, permanent waterway is ca. 2.4 km long, ca. 4 ha in surface area, and encircled by a paved path that lies within 10 m of the water’s edge. Pedestrians, cyclists, and maintenance vehicles commonly use this path, and runoff from the UC Davis campus and the City of Davis discharges into the waterway—all serving as potential sources for plastic pollution. A native population of Northwestern Pond Turtles, *Emys marmorata* and a non-native population of Red-eared Sliders, *Trachemys scripta elegans* inhabit the UCD Arboretum. Red-eared Sliders have been introduced across the western United States and worldwide, primarily due to abandonment of unwanted pets^29^. Illegal release of pet turtles likely founded and continues to supplement the population of *T. s. elegans* in the UCD Arboretum. More detailed site descriptions, maps, and past turtle research in this system are available elsewhere^30–33^.

### Sample collection and analysis

We trapped for *T. s. elegans* from 27 May–2 June and from 24–30 September, 2012. The May/June trapping was part of an unrelated experiment assessing body condition change in *E. marmorata* following prior *T. s. elegans* removal efforts^32^. In decreasing order of frequency, we captured turtles using dipnets, submersible traps, hoop nets, by hand, and in a basking trap. Using multiple capture techniques likely minimized demographic or behavioral biases in our population sample. Within four hours of capture (and usually immediately after capture), we recorded each turtle’s mass using a digital pan scale to the nearest 0.1 g, and measured straight-line carapace length (CL) to the nearest 0.1 mm using dial calipers. We assumed that mass error was a function of turtle size (2% of the mass value), and also assumed a fixed error of 0.1 mm for CL. We euthanized each *T. s. elegans* with an overdose of sodium pentobarbital, followed by fixation in a 10% volume dilution of 37% formalin, and final preservation in 70% ethanol. We deposited these specimens at the Natural History Museum of Los Angeles County (LACM 190504–190568). Our work was authorized under California Department of Fish and Wildlife Scientific Collecting Permit No. 4307, and University of California Davis Institutional Animal Care and Use Committee Protocol No. 16227; we performed all animal handling and collection in accordance with these relevant guidelines and regulations. Our study also complied with the ARRIVE 2.0 guidelines.

We later dissected these turtle specimens by using a Dremel tool to cut across the carapace/plastron bridge, and a scalpel to free the plastron from soft tissue. We then isolated the stomach, and used dissecting scissors to slit it between the esophageal and pyloric sphincters and expose the contents. We carefully transferred all contents to a petri dish using forceps, sorted them under a 6X dissecting microscope, visually identified suspected plastic debris based on texture and color, and stored this debris in separate shell vials labeled with the corresponding LACM catalog number. We also used dissecting scissors to slit the intestinal tract between the pyloric sphincter and the colon, and processed all exposed intestinal contents using this same methodology. Our microscopy-based detection methods readily allowed identification of plastic fragments greater than 0.5 mm in diameter, but prevented us from identifying all but larger microplastic fragments (if present). We did not implement a digestion protocol because doing so would have destroyed diet and parasite material that we intend to study at a later time. We controlled for possible sample contamination by ensuring that our tools were made of metal, and nitrile gloves (when worn) were never damaged during sample processing. After manually cleaning most of the dirt and organic debris from all suspected plastics, we recorded dry mass using a digital pan scale to the nearest 0.0001 g and length along the longest axis using digital calipers to the nearest 0.1 mm.

As is becoming increasingly common in the plastic pollution literature^34–36^, we tested all suspected plastics using Raman spectroscopy, which is non-destructive to samples. Motivating this test was our difficulty in distinguishing true plastic from plastic-resembling organic material such as fragments of crayfish exoskeletons or snail shells, which were common items in the turtles’ gastrointestinal tracts.

We performed Raman spectroscopy on a Horiba XploRa + micro–Raman spectrometer using an incident wavelength of 532 nm, 100 μm slit, 1800 gr/mm diffraction grating and a 10x (0.1 NA) objective, or 100x (0.9 NA) objective when samples appeared to have a coating, were very thin (< 1 mm thickness), or showed a high degree of fluorescence. We initially collected the spectra from 100 cm^−1^ to 3500 cm^−1^ when possible. We performed spectra baseline subtraction and peak fitting using a Gaussian peak shape in the MagicPlot program. To identify materials, we used the SLoPP and SLoPP-E databases^37^ integrated with a custom Python based search/match program (Celestian in prep), together with the RRUFF database^38^.

Lastly, we examined body condition of turtles with and without ingested plastics, using graphical representations and a Mann–Whitney U test performed in R to examine whether those with plastics had lower body conditions than those without plastics.

## Results

### Literature review

Our comprehensive review of turtle dietary literature published prior to 2021 revealed that all 7 marine turtle species (100%) have been documented ingesting plastic^5^. In contrast, only 5 of 352 non-marine turtle species (1.4%) have been documented ingesting plastic^23,39–43^. All five are aquatic species (Table S1). Turtle ingestion of synthetic fishing line is also frequently reported^44–46^, but because this fishing line is likely ingested inadvertently when turtles take baited fish hooks or lures^47,48^, we consider this a very different form of plastic pollution and do not discuss these records further. Additionally, we identified eight non-marine turtle species that have been reported ingesting non-plastic litter^23,41–43,49–58^. These eight include both aquatic and terrestrial species (Table S1).

Our review of marine and non-marine turtle dietary research published within the past 10 years identified 219 turtle diet papers in which plastic ingestion was, or potentially could have been, documented. Of these, 107 (48.9%) involved solely marine turtles, despite these turtles comprising only 2% of all turtle species. Marine turtles thus averaged 15.3 dietary studies per species during this period, whereas non-marine turtles averaged 0.3 dietary studies per species.

Plastic and litter ingestion literature for non-marine turtles was generally challenging to identify. Terms like “plastic,” “litter,” and “trash” were always absent from the title, abstract, and key words, and were sometimes mentioned only in tables. Discovery of these data thus required examination of diet tables and/or relevant parts of the text. Additionally, authors occasionally made vague mention of possible plastic, reporting “inorganic remnants,” “litter,” “manmade debris,” or “manufactured items” consumed by turtles without elaborating further^59–64^.

### Field survey and plastic confirmation

We captured and euthanized a total of 65 *Trachemys scripta elegans* from the UCD Arboretum, including 29 hatchlings (CL 32–44 mm), 25 juveniles (CL 50–98 mm), 8 adult females (CL 123–180 mm), and 3 adult males (CL 100–160 mm; size classes follow Ernst and Lovich, 2009^58^). The strong representation of young turtles in our sample is likely attributable to an earlier project that removed most of the adult *T. s. elegans* from this waterway^32^; the turtle carcasses from that study were unavailable to us, having been either incinerated or used for teaching dissections. Following recent recommendations for reporting ingested plastics in marine turtles^20^, we report frequency, quantity, and normalized quantity of ingested plastic debris.

**Figure 1:**
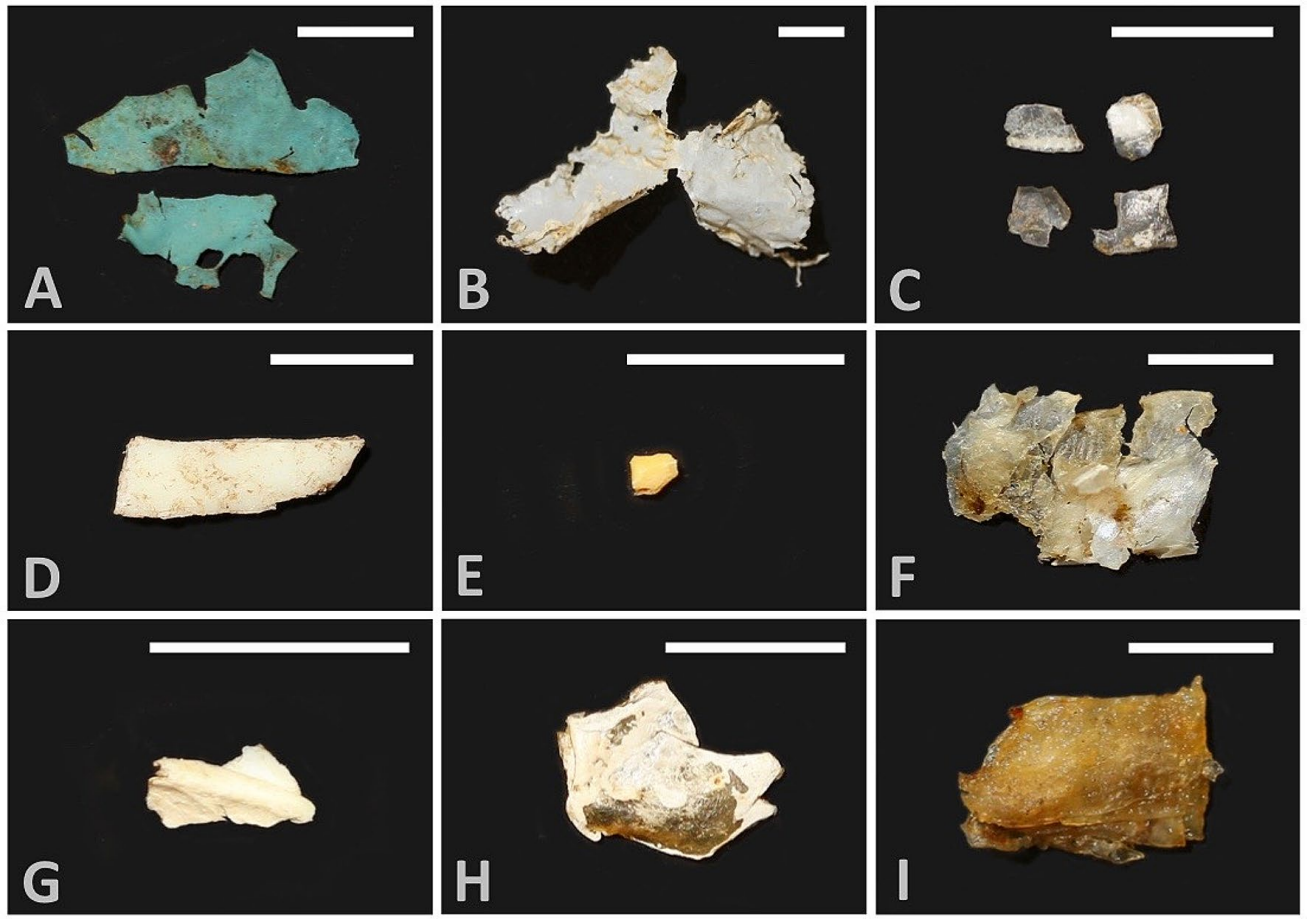
Plastic (**A**–**E**; LACM 190543, 190527, 190550, 190547, 190529) and plastic-resembling organic material (**F**–**I**; LACM 190564, 190560, 190550, 190564) ingested by Red-eared Sliders, *Trachemys scripta elegans*, in the University of California Davis Arboretum, USA. These images illustrate the visual similarity of certain plastics and non-plastics (e.g., panels **C**,**F**,**D,G**) and thus the value of quantitative testing; we tested all fragments shown using Raman spectroscopy. Scale bar = 5 mm in all panels. Some items have the same LACM number because they originated from the same turtle.

Within the gastrointestinal tracts of these 65 *T. s. elegans*, we observed both macroplastics > 5 mm long (n = 4) and microplastics < 5 mm long (n = 5). Five turtles had plastic in their gastrointestinal tract, for a total plastic ingestion frequency of 7.7% (Fig. 1; Table 1). Turtles with ingested plastic comprised one hatchling, three juveniles, and one adult female (Table 1). Two turtles had plastic only in their stomachs, while three turtles had plastic only in their intestines. The ingested plastic comprised 1–4 fragments in each turtle, and each fragment ranged from 1.0–26.3 mm along its longest axis, while total plastic mass in each turtle ranged from 0.0001–0.0225 g (Table 1). Total plastic quantity was 0.0457 g, and normalized plastic quantity was 0.0802 g/kg of turtle body mass. Plastic types included: nitrile likely from a blue exam glove (Fig. 1A), a composite of polyethylene and rutile pigment possibly from a white plastic shopping bag (Fig. 1B), a composite of likely polycarbonate with synthetic orange pigment 36 (Fig. 1C), white polystyrene possibly from disposable cutlery (Fig. 1D), and a composite of likely polyethylene with organic material (Fig. 1E).

**Table 1:** Plastic and non-plastic human litter ingested by Red-eared Sliders, *Trachemys scripta elegans*, in the University of California Davis Arboretum, USA.

Specimen no	Age/sex	Carapace length (mm)	Litter type (stomach)	Litter type (intestine)	Total litter mass (g)	Litter longest axis (mm)
LACM 190543	Juvenile	67.7	Nitrile (2 pieces)	n/a	0.0047	18.4
LACM 190527	Female, adult	132.9	n/a	Composite, see text (1 piece)	0.0225	23.6
LACM 190550	Juvenile	64.3	n/a	Composite, see text (4 pieces)	0.0003	2.7
LACM 190547	Juvenile	66.4	Polystyrene (1 piece)	n/a	0.0135	7.8
LACM 190529	Hatchling	32.6	n/a	Composite, see text (1 piece)	0.0001	1.0
LACM 190548	Juvenile	75.3	White paper (1 piece)	n/a	0.0046	8.5

**Figure 2:**
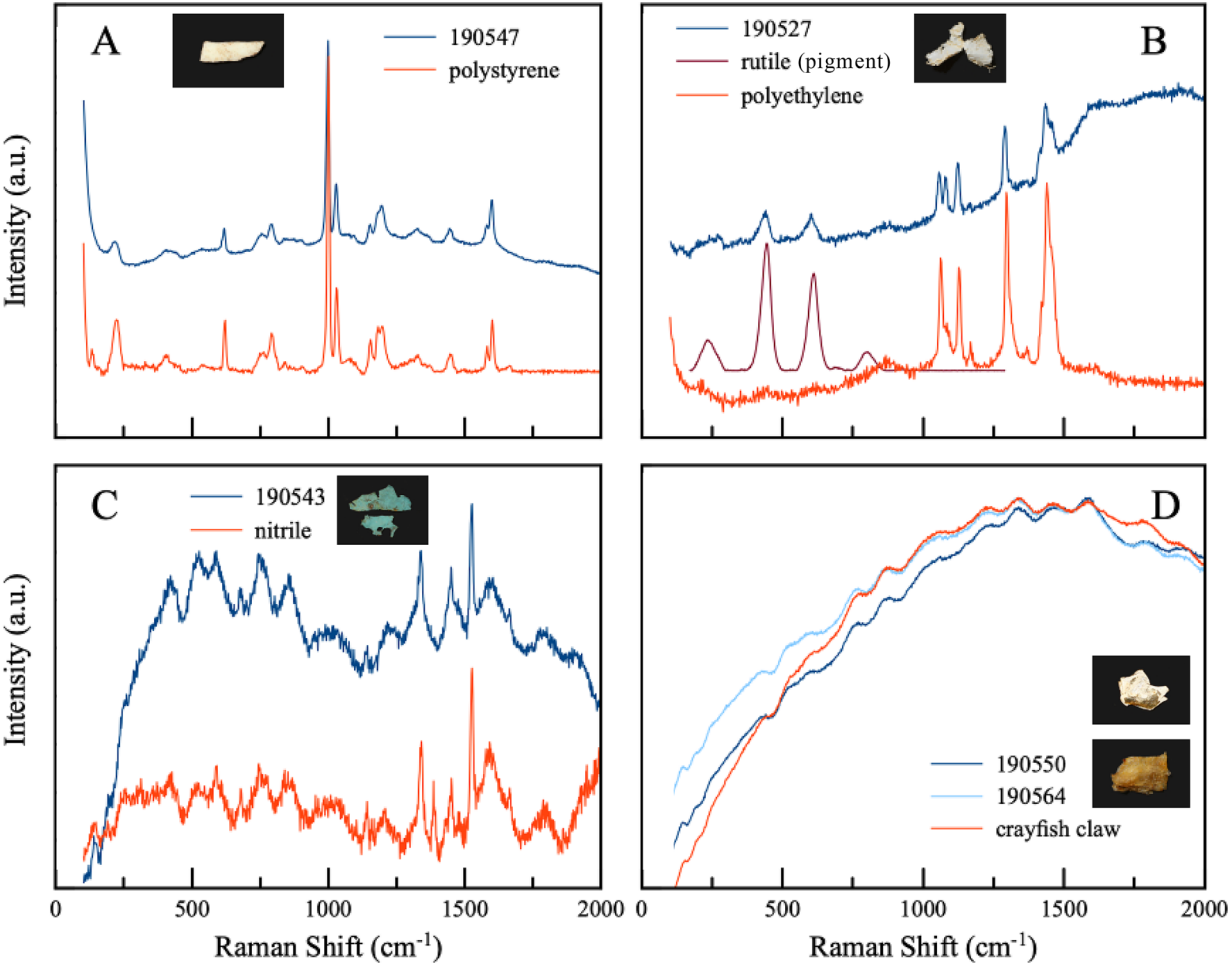
Characterization of plastic and plastic-resembling organic material ingested by Red-eared Sliders, *Trachemys scripta elegans*, from the University of California Davis Arboretum, USA. Each panel shows Raman spectra from diet sample(s) in blue and known reference material(s) in orange or dark red. In each panel, spectra are vertically offset for improved visualization, and hence the y-axis is in terms of arbitrary units (a.u.). The horizontal position of the spectral peaks is the relevant metric for confirming sample/reference match in each panel. Sample numbers correspond to LACM catalog numbers, and inset photos show the exact diet samples tested (refer to Fig. 1).

We isolated material from the stomach and/or intestine of two additional turtles (plus one turtle with confirmed plastic: LACM 190550) that we suspected was plastic based on color and texture (Fig. 1E–H). However, Raman spectroscopy confirmed that all of these fragments were actually organic material. Figure 2 presents Raman spectra for selected plastics and plastic-resembling organics.

Additionally, another turtle’s stomach contained a fragment of white paper with a waxy coating possibly from a disposable drinking cup (Table 1), highlighting that turtles in this system also consume non-plastic human litter. This paper fragment did not produce a usable Raman spectra. Nonetheless, we are confident in its identify because the white color, densely fibrous nature, and waxy surface texture of this two-dimensional fragment was both consistent with paper and unique among all turtle dietary items we found.

There was no signal of plastic ingestion negatively affecting turtle body condition. Regression of mass against carapace length showed that turtles with ingested plastic lay within or above the 95% confidence interval of the regression line for turtles without plastic (Fig. S1), and mass divided by carapace length was not significantly different between turtles with and without ingested plastic (Fig. S2; W = 123, *p* value = 0.5142). All ingested plastics were integrated within or at the margin of a food or fecal bolus instead of being isolated, and none were physically imbedded in the wall of the gastrointestinal tract. No ingested plastic appeared to be blocking material transfer within the gastrointestinal tract.

## Discussion

Our literature review and field study underscore that non-marine turtles are ingesting plastic and non-plastic litter, but research output on turtle diet, and therefore studies that could identify plastic ingestion, is heavily biased toward marine species. Among non-marine turtles, at least 5 of 352 non-marine turtle species are known to ingest plastic. In marine turtles by comparison, all 7 species are known to ingest plastic. Furthermore, nearly 50% of recent, relevant dietary studies involve this small component of global turtle diversity, and in the last 10 years marine turtles averaged over 50 times more dietary studies per species than non-marine turtles. Additionally, the first of five global review articles on plastic ingestion by marine turtles was published 35 years ago^65^, but no such reviews exist for non-marine turtles. Cumulatively, these results highlight that plastic ingestion has long been understudied in non-marine turtles relative to marine species, and that plastic ingestion remains unassessed or poorly assessed in many non-marine turtle species. Additional studies are needed to determine if plastic ingestion poses a greater threat to non-marine turtles, and to other freshwater taxa, than is currently understood. The need for such studies will become only more pressing as plastic pollution continues to accumulate^1^.

There are several possible explanations for the comparative lack of scientific publications on plastic and human litter in non-marine turtle diets. As indicated by our review, studies that report plastic or human litter ingestion by turtles do not flag these results in their title, abstract, or key words, thus reducing their visibility to readers. Extensive stranding and salvage networks for marine turtles, plus high public awareness of marine turtle endangerment, likely increases opportunities for researchers to examine dead or ill marine turtles relative to non-marine turtles. Furthermore, ingested plastic might be harder to detect in non-marine turtles because they generally have smaller body sizes compared to marine turtles, thus reducing the likelihood of non-marine turtles ingesting large, easily identified plastic fragments. Conversely, this imbalance might also result from plastic ingestion being truly more common in marine turtles. Marine plastic pollution volume might be greater overall than in non-marine systems, and all marine turtles, but especially the Leatherback *Dermochelys coriacea*^66^, will eat sea jellies and are thus prone to mistaking plastic bags for prey^18^. Regardless of the cause(s) of this imbalance, our work should motivate greater researcher investment into non-marine turtles and other at-risk freshwater species to better evaluate whether plastic ingestion is affecting these already extraordinarily imperiled animals.

Different study methodologies coupled with environmental factors can influence reported plastic ingestion frequencies in marine turtles^24^, and we expect the same is true of non-marine turtles. Importantly, our field study is one of few that includes only turtles captured alive, as opposed to turtles found dead or dying. Although our search for studies reporting such comparatively unbiased samples is non-comprehensive for marine turtles, the reported plastic ingestion frequencies we identified varied widely, ranging from 4%^67^ to 76%^68^. For non-marine turtles, only three plastic ingestion frequencies are available. A plastic ingestion frequency of 21.6% is reported for *Graptemys flavimaculata*^40^, while frequencies of 32.0%^43^ and 40.6% ^23^ are reported for *Trachemys scripta elegans* and *T. dorbigni*, respectively. However, the frequencies of the latter two studies are inflated to an unknown degree, because they combine plastic with other human litter and debris (paper and/or stones). Additionally, no studies on plastic ingestion by non-marine turtles chemically verified or characterized their reported plastics.

Raman spectroscopy was critical to accurately assessing plastic ingestion frequency in our turtle samples. Without this quantitative plastic verification, our reported ingestion frequency could have been artificially inflated from 7.7% to 10.8%, constituting a 40% increase. We recognize that many plastic fragments, such as those that are large or brightly colored, are nearly impossible to mistake for organic debris. However, other fragments can be harder to positively identify. This is especially true for non-marine turtles, because as mentioned earlier, their generally smaller body sizes relative to marine turtles increases the likelihood that ingested plastics will be small, less easily identified fragments. Our results underscore the value of quantitative verification when studying plastic ingestion by small-bodied animals.

Based on our total sample size of 65 *T. s. elegans*, our data suggest that plastics are not accumulating in the stomachs of turtles in the UCD Arboretum, but rather are passing through the gastrointestinal tract. Furthermore, based on our sample of 29 hatchling turtles, this age class seems to be ingesting plastics at a much lower rate than other age classes. Additionally, we found no indication of turtle body condition being negatively affected by plastic ingestion. Although our study thus offers no clear evidence that plastic ingestion by *T. s. elegans* in this system is causing gastrointestinal impaction or weight loss, it is possible that those effects are happening and went undetected. We also did not test whether plastic ingestion is resulting in sub-lethal physiological stress to turtles in this population.

The transferability of our results to other non-marine turtle populations is unclear. Although our sample involves turtles from only a single site, our sample size exceeds that of 70% of plastic ingestion studies on marine turtles^20^. Our literature review further underscores the almost complete lack of comparable data in non-marine turtles. Greater scientific commitment to the study of plastic ingestion across the diverse, globally distributed, and highly imperiled non-marine turtle assemblage is thus urgently needed.

Although our UCD Arboretum study was restricted to a non-native turtle, it is potentially a useful proxy for how plastic pollution affects native turtles. In our specific case, the co-occurring native turtle is the Northwestern Pond Turtle, *Emys marmorata*, which is listed as Endangered in Washington state^69^, Sensitive-Critical in Oregon^70^, and a Species of Special Concern in California^71^. Together with its sister taxon *E. pallida*^72^, *E. marmorata* is also currently undergoing a Status Review for possible listing under the federal Endangered Species Act^25^. Due to these conservation concerns, lethal sampling of *E. marmorata* or *E. pallida* for gastrointestinal tract dissections is inadvisable, and invasive methods like stomach flushing are also potentially problematic—making the proxy method appropriate. Although *T. s. elegans* and *E. marmorata* show some spatial and behavioral segregation in the UCD Arboretum^31,32^, both species co-occur throughout the waterway and are roughly comparable in body size^58^. Furthermore, both *T. s. elegans* and *E. marmorata* are dietary generalists, and both species shift to more herbivorous diets as adults, although this shift is much more pronounced in *T. s. elegans*^58^. For these reasons, our observed plastic ingestion by *T. s. elegans* suggests that plastic is being similarly ingested by co-occurring *E. marmorata*.

We encourage replication of our study elsewhere in the range of *E. marmorata* and *E. pallida*, to better evaluate if plastic ingestion is a relevant environmental concern. Such replication is feasible because non-native *T. s. elegans* are established at many sites inhabited by these two at-risk native species^73,74^. Our proxy approach is also globally relevant because non-native *T. s. elegans* co-occur with populations of many endangered native turtle species, being widely established on every continent except Antarctica^29^. Additionally, because unwanted pet *T. s. elegans* are more commonly released into urban waterways due to proximity to humans, this species is especially likely to occur in habitats where they encounter abundant plastic litter. In fact, the semi-urbanized character of the UCD Arboretum likely made it a particularly relevant site for studying plastic ingestion. Worldwide, five of seven studies that report plastic ingestion by non-marine turtles (including ours) involve animals from urbanized habitats. This pattern suggests that turtle populations in urbanized waterways could be especially relevant for future research on plastic ingestion.

Some of the juvenile and adult *T. s. elegans* in our field sample may have been recently-released pets, which could have affected our results in two opposing ways. First, some ingested plastics could be a holdover from captive conditions, rather than reflecting plastic ingestion in the UCD Arboretum. Alternatively, captive turtles may have been plastic-free when released into the UCD Arboretum, and were then captured by us before sufficient time had passed for them to ingest available plastic pollution. Although it is difficult to estimate the magnitude of these potential biases because data are unavailable on frequency of turtle introductions at this site, we consider it unlikely that they meaningfully affected our results.

Some ingested plastic that we documented in our *T. s. elegans* field sample may also have been attributable to trophic transfer (i.e., turtles eating prey that had itself ingested or become entangled in plastic). Trophic transfer was recently reported in a riverine bird^9^ and potentially in some freshwater fishes^75^. For certain sea turtle species, eating jellyfish or seagrass with ingested/entangled plastic could facilitate trophic transfer^35,76^, as could eating benthic bivalve mollusks in marine and freshwater systems. For two *T. s. elegans* in our sample, crayfish that we documented in their gastrointestinal tract may have been the source of the dietary plastic we observed, although this is speculative.

Habitat degradation and overexploitation are recognized as the leading threats to global turtle diversity^17^. We concur with this assessment, and recognize the need to focus on drivers of declines. Unfortunately, turtles also face many other threats. A full accounting of those threats, including the potential threat posed by plastic ingestion, is necessary for informed conservation efforts. One emerging threat to freshwater turtles that, like plastic pollution, has been generally overlooked is ingestion of fishhooks and lures^47,48^. This inattention has come despite simulation modeling showing that ingested fishhooks alone could cause population declines in multiple turtle species^48^. Increased recognition of all potential stressors on turtles, and on other freshwater animals in the case of plastic pollution, remains important.

In the context of plastics, there is a pressing need to explore both the potential for turtles being selective in how they ingest plastic pollution^24^, and the potential effects of microplastic ingestion. Such studies have only just begun to appear for marine turtles^19,77,78^, and these topics remain unexamined in wild freshwater turtles. Understanding if dietary plastic selectivity exists is particularly relevant for informing management interventions that could target plastic types most attractive to turtles.

### Recommendations for increased study and reporting of plastic ingestion research involving freshwater animals

As a call to action, we invite the freshwater biology community, and particularly those who study and manage freshwater turtles, to prioritize acquisition and publication of plastic ingestion data. Furthermore, we emphasize the need for consistent reporting methods and terminology. To achieve these goals, we offer the following best-practice recommendations.

First, we encourage replication of our study by conservation practitioners who lethally remove non-native turtles from the wild. We urge that such removals be recognized as a golden opportunity for using non-native turtles as a proxy for how native, declining turtles might be affected by plastic pollution. Many removals of *T. s. elegans* are ongoing worldwide^79^, thus offering clear potential for rapidly implementing this recommendation. Given the severe imperilment of many non-marine turtle species, such proxy studies may be among the only defensible ways to document the scope of plastic ingestion.

Second, we encourage permitting agencies to require that non-native turtles lethally removed from the wild be deposited in a reputable museum collection (see similar recommendations elsewhere^80^). We appreciate that it may be unfeasible to deposit all removed turtles in museum collections, due to logistical reasons including storage space and specimen preparation burdens. However, depositing at least a representative sample of removed turtles in museum(s), with the collectors being responsible for at least some of the associated logistical and financial burdens (i.e., helping prepare specimens and providing funds for preparation and storage costs), should be a regulatory requirement. We also recognize that managers who remove non-native turtles may lack the resources to implement plastic dissection studies themselves. Thus, requiring that specimens be deposited in museums will ensure that they can be later studied by others not only for plastic ingestion, but also parasite loads, reproductive condition, and other useful data that can inform broader conservation and management goals.

Third, we encourage veterinarians, field biologists, and others who examine recently dead turtles to dissect their gastrointestinal tract to determine plastic presence/absence, and to publish their findings as discussed below. Euthanizing native turtles for comprehensive gastrointestinal tract examination is usually inadvisable due to conservation concerns. Additionally, less invasive fecal analysis and stomach flushing techniques could lead to underreporting of plastic ingestion frequency and severity, because plastic that is stuck in the gastrointestinal tract would likely go undetected. Given these and other limitations, anecdotal observations from dead turtles will remain critical for improving scientific understanding of the geographic, ecological, and taxonomic scope of plastic ingestion by non-marine turtles.

Fourth, we encourage the use of standardized methods and terminology for any peer-reviewed study reporting plastic ingestion. All plastic ingestion data for non-marine turtles that we found was unflagged in the publication title, abstract, and key words, and was often hidden in diet tables and/or excluded from discussion. These factors made it extremely challenging to identify the current state of knowledge on plastic ingestion in turtles. Fragments of invertebrate exoskeletons, shell, and bone can also be easily mistaken for plastics, and this risk should be mitigated using appropriate techniques. Additionally, frequency and quantity of plastic ingestion is also rarely reported in non-marine turtle diet studies. To remedy these interrelated problems, we encourage the following:

1. Always include “plastic” in the title, abstract, or key words for any publication that contains data on plastic ingestion.
2. Quantitatively confirm plastics and/or deposit suspected dietary plastic in a museum collection. We recognize that commonly-used quantitative tools like Raman spectroscopy or FT-IR spectroscopy are not available to everyone; thus, it is imperative that presumed plastics be deposited in a reputable museum collection to enable future confirmatory testing.
3. Report frequency, quantity, and normalized quantity of ingested plastics whenever possible, following prior recommendations^24^.
4. Publish plastic-ingestion data in widely read, indexed outlets. For anecdotal notes or short communications of turtle dietary plastic, we highlight *Herpetological Review*, *Chelonian Conservation and Biology*, and *Herpetology Notes* as scientific journals with broad readership among the turtle community that have minor or no publication fees.

We emphasize that our best-practice recommendations are equally relevant for studies of non-plastic human litter ingestion by turtles. Although plastics receive more scientific attention, ingestion of metal, glass, paper, and many other types of human debris is happening (Table S1) and can have similar negative effects on animals. This litter is thus equally important to document in accordance with our recommendations. We further underscore that publishing negative data (i.e., dietary studies that detect no ingested plastic or human litter) is equally as important as publishing positive data.

The scientific community remains far from a representative understanding of the environmental impacts of plastic pollution in non-marine ecosystems. Our work showcases this knowledge gap as it relates to non-marine turtles—just one of many susceptible freshwater animal groups of global conservation concern. We invite others to answer our call for greater research investment on this theme, and hope that our recommendations offer a roadmap for achieving this goal.

## Supplementary Information

Supplementary Information

## Data Availability

The datasets generated during the current study are available from the corresponding author on reasonable request.
