# Supplementary material for: Plastic ingestion by freshwater turtles: a review and call to action
Source: Sci Rep. 2021 Mar 23;11:5672. doi: 10.1038/s41598-021-84846-x (PMC7987988; doi:10.1038/s41598-021-84846-x)
Supplement: Supplementary file 1 — Supplementary Information [file 41598_2021_84846_MOESM1_ESM.docx]

**Plastic ingestion by freshwater turtles: A review and call to action**

Adam G. Clause^1^, Aaron J. Celestian^2^, and Gregory B. Pauly^1^*

^1^Urban Nature Research Center & Department of Herpetology, Natural History Museum of Los Angeles County, Los Angeles, California, USA

^2^Department of Mineral Sciences, Natural History Museum of Los Angeles County, Los Angeles, California, USA

*Corresponding author (email: gpauly@nhm.org)

**SUPPLEMENTARY INFORMATION**


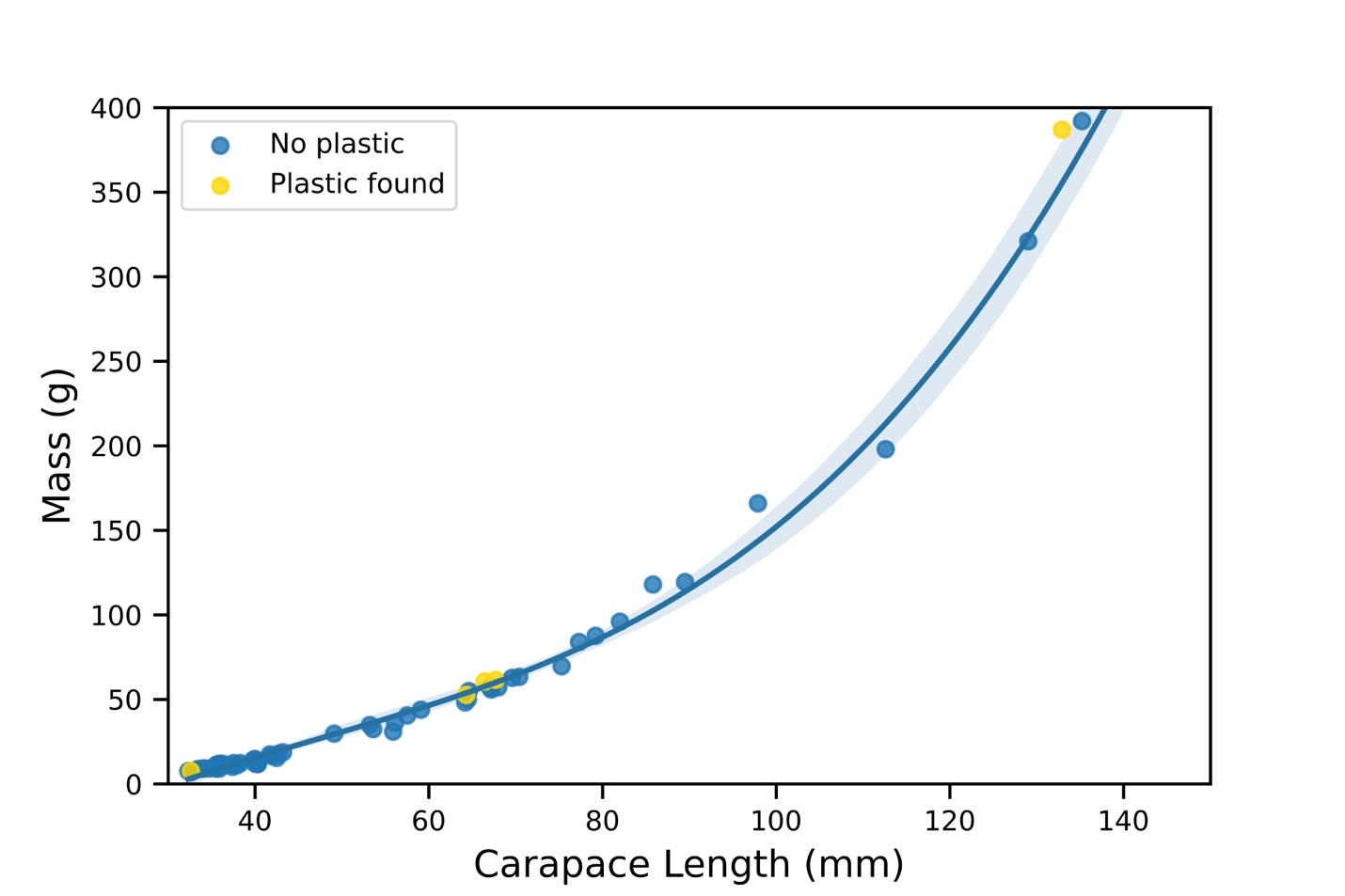


**Figure S1.** Body condition (mass regressed against straight-line carapace length) of Red-eared Sliders, *Trachemys scripta elegans*, from the University of California Davis Arboretum, USA. Pale blue shading indicates the 95% confidence interval of the regression line for all turtles without ingested plastic (n = 60), but only turtles with carapace length less than 140 mm are shown (n = 58).

**Figure S2.** Boxplot comparing the log of body condition (mass divided by straight-line carapace length) of Red-eared Sliders, *Trachemys scripta elegans*, from the University of California Davis Arboretum, USA that did nor did not ingest plastic. Black dots represent values of individual turtles, and are horizontally jittered for improved visualization.

**Table S1.** Studies reporting ingestion of plastic (***bold italic***) and non-plastic human litter by non-marine turtles.

| **Family** | **Species** | **Ecology** | **Country** | **Litter Type(s)** | **Source** |
| --- | --- | --- | --- | --- | --- |
| Chelidae | *Chelodina longicollis* | Aquatic | Australia | Metal | Meathrel et al. 2004 |
| Chelydridae | *Macrochelys temminckii* | Aquatic | USA | Glass, metal, thread | Elsey 2006 |
| Chelydridae | *Macrochelys temminckii* | Aquatic | USA | Cardboard | Sloan et al. 1996 |
| Chelydridae | *Macrochelys temminckii* | Aquatic | USA | Rubber | Iverson pers. comm. (Ernst & Lovich 2009) |
| Emydidae | *Chrysemys picta picta* | Aquatic | USA | ***Styrofoam***, glass | Padgett et al. 2010 |
| Emydidae | *Graptemys flavimaculata* | Aquatic | USA | ***Polystyrene*** | Selman & Lindeman 2018 |
| Emydidae | *Malaclemys terrapin rhizophorarum* | Aquatic | USA | Glass | Denton et al. 2015 |
| Emydidae | *Malaclemys terrapin* | Aquatic | USA | Cigarette filter | Outerbridge et al. 2017 |
| Emydidae | *Terrapene carolina* | Terrestrial | USA | Paintball | Walde & Christensen 2007 |
| Emydidae | *Terrapene carolina bauri* | Terrestrial | USA | Paper | Platt et al. 2009 |
| Emydidae | *Trachemys dorbigni* | Aquatic | Brazil | ***Plastic***, paper | Silveira et al. 2019 |
| Emydidae | *Trachemys scripta elegans* | Aquatic | China | ***Plastic***, cloth, paper | Wang et al. 2013 |
| Emydidae | *Trachemys scripta elegans* | Aquatic | France | ***Plastic***, paper | Prévot-Julliard et al. 2007 |
| Emydidae | *Trachemys scripta elegans* | Aquatic | USA | ***Polyethylene***, ***polystyrene***, ***nitrile***, paper | This work |
| Geoemydidae | *Batagur trivittata* | Aquatic | Myanmar | ***Plastic*** | Kuchling & Lwin 2004 |
| Testudinidae | *Gopherus agassizii* | Terrestrial | USA | Balloon, ribbon | Walde et al. 2007 |
| Testudinidae | *Gopherus agassizii* | Terrestrial | USA | Glass, foil | Burge 1989 |
